# Localized Tonsillar Amyloidosis Associated With a Plasma Cell Neoplasm Presenting as an Oropharyngeal Mass: A Diagnostic Challenge

**DOI:** 10.7759/cureus.107877

**Published:** 2026-04-28

**Authors:** José Gonzalo Bravo Quiroz, Hillary Lizarraga, José Manuel García Romero, Maria Paula Morales Ruiz, Miguel Alfredo Gracía De La Cruz

**Affiliations:** 1 Otolaryngology - Head and Neck Surgery, Hospital General Dr. Manuel Gea González, Mexico City, MEX; 2 General Surgery, Universidad Autónoma de Querétaro, Santiago de Querétaro, MEX; 3 Plastic Surgery, University Hospital Coventry and Warwickshire, Coventry, GBR

**Keywords:** extramedullary plasma cell neoplasm, lambda-restricted plasma cell neoplasm, oropharyngeal mass, surgical case reports, tonsillar amyloidosis

## Abstract

Amyloidosis is a rare and heterogeneous group of disorders characterized by extracellular deposition of misfolded proteins, which may present as either localized or systemic disease. In the head and neck region, the presentation of amyloidosis is uncommon and may clinically mimic neoplastic processes.

We report the case of a 58-year-old man with a five-year history of progressive oropharyngeal symptoms, initially perceived as a foreign body sensation and intermittent odynophagia, later evolving to dysphagia to solids. Physical examination revealed marked asymmetry of the palatine tonsils due to a mass originating from the left tonsillar fossa. Imaging studies confirmed a well-defined lesion without clear signs of invasion. Given the progressive symptoms and the size of the lesion, surgical excision was performed.

Histopathological analysis demonstrated amyloid deposition confirmed by Congo red staining, with immunophenotypic features consistent with a lambda-restricted plasma cell neoplasm. The patient was referred for further hematologic evaluation to exclude systemic disease, but declined additional workup.

This case illustrates how localized amyloidosis may closely resemble neoplastic disease both clinically and radiologically, and highlights the central role of histopathological evaluation. It also emphasizes the importance of considering systemic involvement even in apparently localized presentations.

## Introduction

Amyloidosis comprises a group of disorders characterized by extracellular deposition of insoluble fibrillar proteins arranged in a β-pleated sheet configuration, leading to progressive tissue dysfunction [1]. These deposits are typically identified histologically by Congo red staining and apple-green birefringence under polarized light [2].

The disease is currently classified by the biochemical nature of the precursor protein, with immunoglobulin light-chain amyloidosis (AL) being the most common systemic form in developed countries [2,3]. In AL amyloidosis, a clonal population of plasma cells produces misfolded light chains that deposit in tissues, either locally or systemically [3].

Localized amyloidosis differs from systemic forms in that amyloid deposition is confined to a single organ, often due to local production of amyloidogenic proteins by plasma cells within the affected tissue [1,4]. Despite this distinction, both entities may share similar clinical presentations, making differentiation challenging without histopathological and systemic evaluation [4].

In the head and neck region, amyloidosis most frequently involves the larynx, while involvement of the oral cavity and pharynx is considerably less common [1-6]. Tonsillar involvement is particularly rare, with only a limited number of cases described in the literature, estimated at fewer than 50 reported cases worldwide [7]. Importantly, localized AL amyloidosis in this region is frequently associated with a local clonal plasma cell proliferation within the affected tissue, a relationship that carries diagnostic and prognostic implications distinct from those of systemic disease [4,7]. In such cases, the clinical presentation may closely resemble more common conditions, including malignancy, which often guides initial diagnostic and therapeutic decisions.

We present a case of localized amyloidosis involving the palatine tonsil, initially approached as a neoplastic process due to its clinical and radiological characteristics. While tonsillar amyloidosis is exceptionally rare, its potential to closely mimic malignancy represents a critical and underrecognized diagnostic challenge. This case addresses a knowledge gap regarding the atypical presentation and clinical course of tonsillar amyloidosis associated with a plasma cell neoplasm, and underscores the indispensable role of histopathological assessment in reaching an accurate diagnosis.

## Case presentation

A 58-year-old man, with no relevant family history, employed as a farmer, and a remote history of tobacco use (10 pack-years, discontinued 20 years before), presented with a long-standing history of oropharyngeal discomfort.

The patient reported that his symptoms began approximately five years prior to evaluation, initially as a persistent foreign body sensation in the oropharynx accompanied by intermittent odynophagia. Over time, the symptoms gradually progressed to dysphagia with solid foods and persistent postnasal discharge. Notably, the patient denied systemic symptoms such as fever, unintentional weight loss, night sweats, dyspnea, or dysphonia, which initially suggested a benign or indolent etiology.

On physical examination, a marked asymmetry of the palatine tonsils was observed, with grade I hypertrophy on the right and significant enlargement on the left (Brodsky grade IV), secondary to a mass originating from the left tonsillar fossa. The lesion appeared pink, without ulceration or evident increased vascularity, which made an overtly malignant lesion less likely on initial inspection. Nevertheless, the combination of marked unilateral tonsillar enlargement, progressive symptom evolution, and extension toward the hypopharynx collectively represented a constellation of findings more commonly associated with neoplastic disease, making this case particularly challenging to differentiate from malignancy on clinical grounds alone.

**Figure 1:**
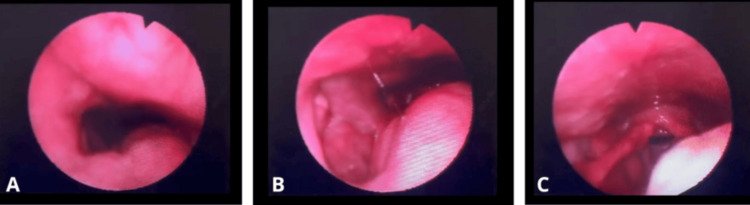
Flexible nasofibrolaryngoscopy findings (A) Endoscopic view of the left oropharyngeal mass from the nasopharynx. (B) Endoscopic view of the left oropharyngeal mass from the oropharynx. (C) Endoscopic view of the left oropharyngeal mass from the hypopharynx

Flexible nasofibrolaryngoscopy demonstrated extension of the lesion toward the hypopharynx, reaching the level of the free edge of the epiglottis, partially obscuring visualization of the posterior pharyngeal wall. Despite this extension, there were no signs of airway compromise (Figure 1).

**Figure 2:**
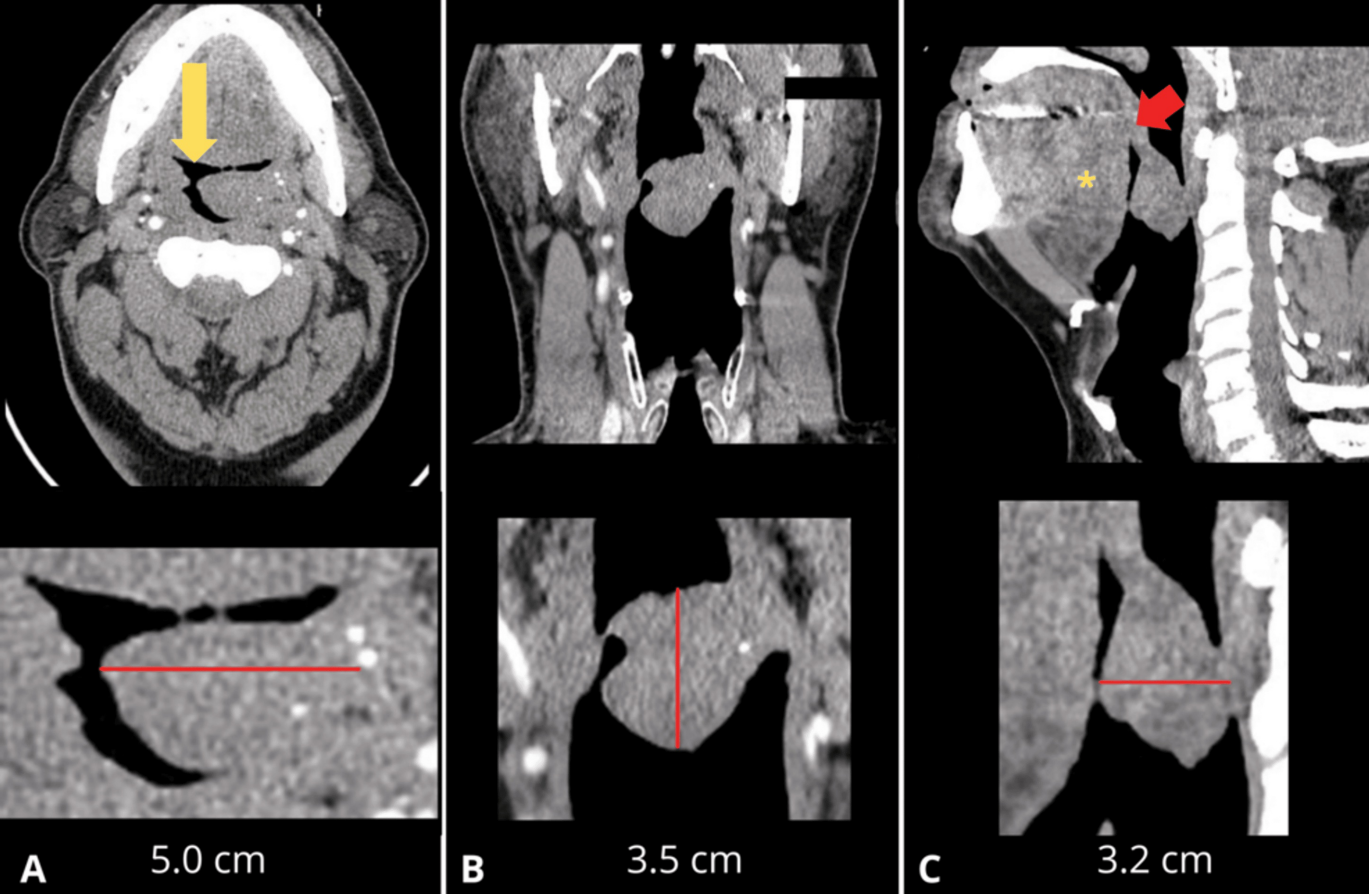
Contrast-enhanced computed tomography showing the left oropharyngeal tumor (A) Sagittal view. (B) Coronal view. (C) Axial view. A well-defined, saccular-appearing mass originates from the anterior wall of the oropharynx (red arrow), measuring 5.0 cm in the axial plane, 3.5 cm in the coronal plane, and 3.2 cm in the sagittal plane. The lesion occupied the left tonsillar fossa and extended medially into the oropharyngeal lumen, resulting in approximately 60%-70% narrowing of the parapharyngeal airway column (yellow arrow). Inferiorly, the mass was in close anatomical relationship with the floor of the tongue (yellow asterisk), without evidence of infiltration. The pharyngeal constrictor muscles maintained their normal appearance without signs of compression or invasion, and the carotid space structures, including the internal carotid artery and internal jugular vein, were preserved with no evidence of displacement or encasement. No pathologically enlarged cervical lymph nodes were identified

Routine laboratory tests did not reveal any abnormalities. A contrast-enhanced computed tomography scan of the neck showed a well-defined mass measuring 5.0 × 3.5 × 3.2 cm arising from the left palatine tonsil, without clear evidence of invasion into adjacent structures (Figure 2).

**Figure 3:**
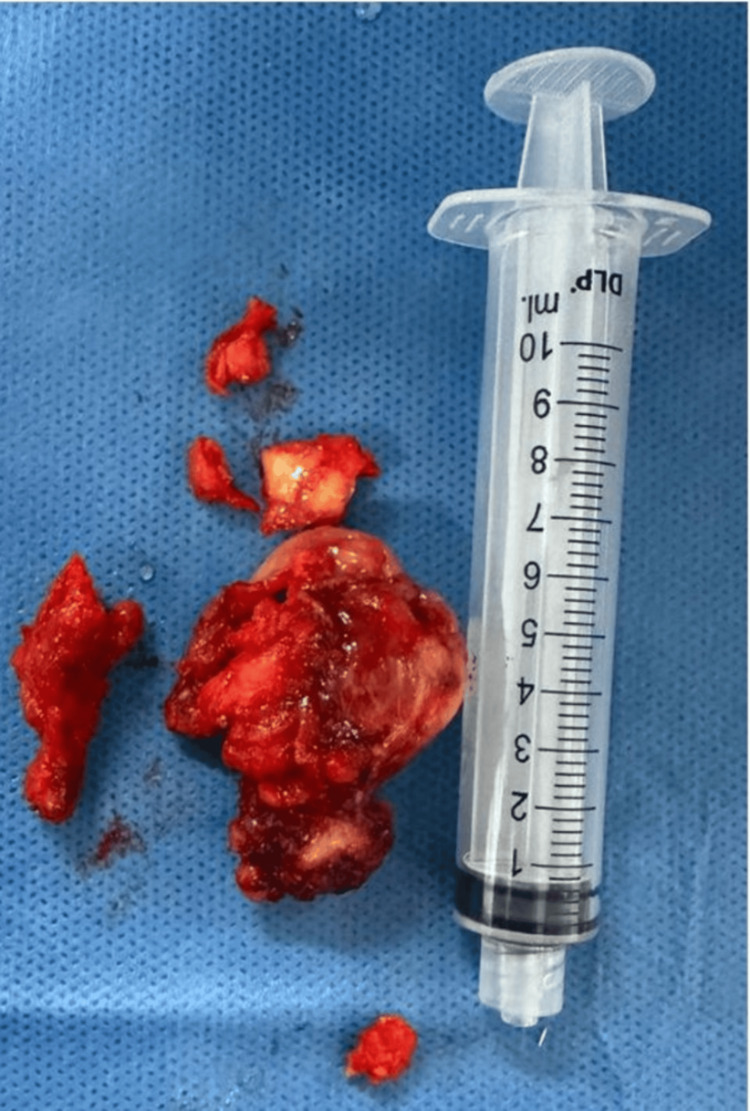
Gross surgical specimen of the resected oropharyngeal mass Gross specimen of the excised left palatine tonsil. The surgical specimen is shown alongside a standard 10-mL syringe (approximately 9.5 cm in length) as a size reference. The specimen measured 5.0 × 3.5 × 3.2 cm as determined by formal pathological examination, consistent with the dimensions reported on preoperative imaging

Given the progressive nature of symptoms, the size of the lesion, and the inability to establish a definitive diagnosis through clinical and imaging findings alone, the decision was made to proceed with surgical excision. A conventional cold dissection tonsillectomy was performed without intraoperative or postoperative complications (Figure 3).

**Figure 4:**
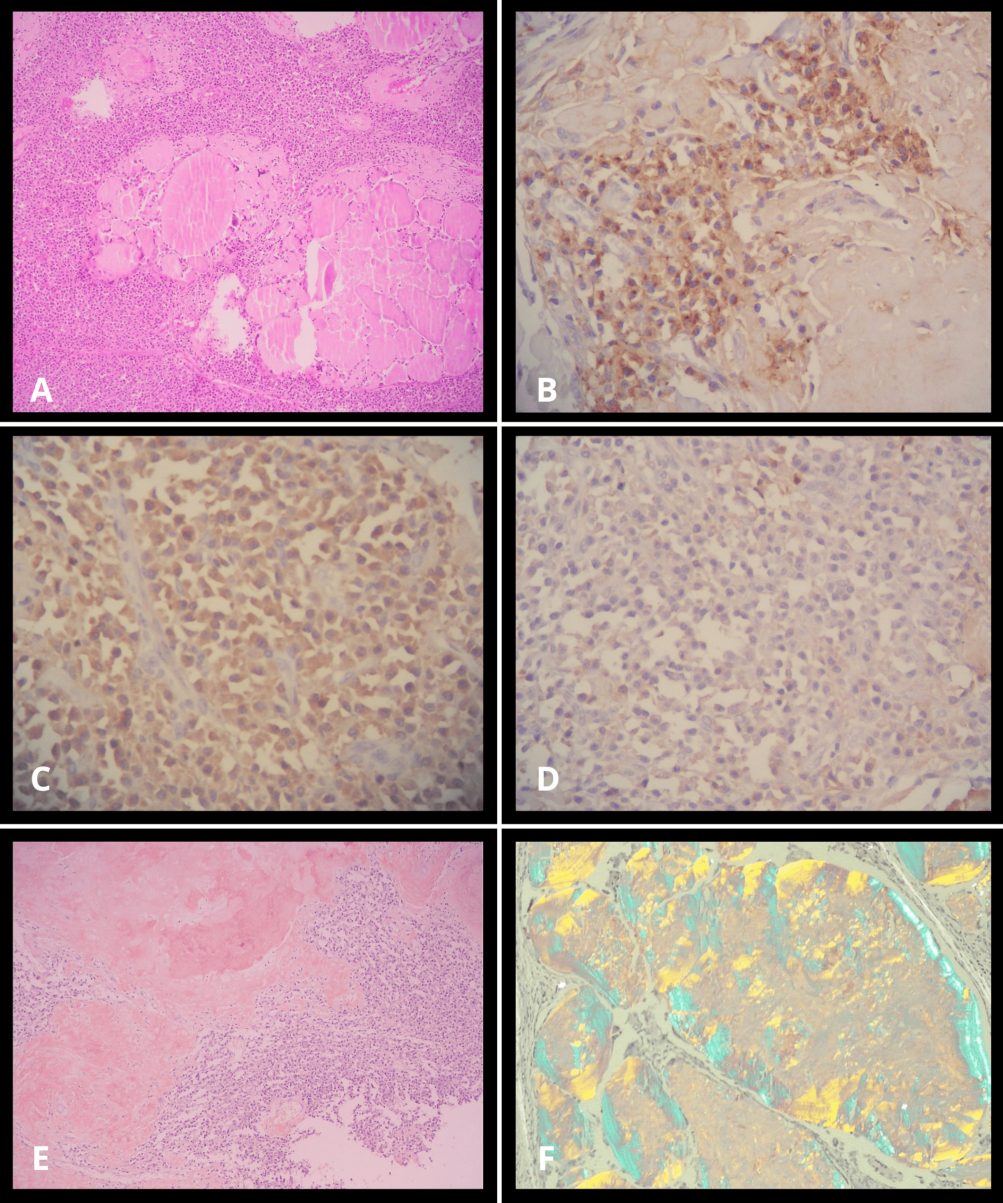
Histopathological findings (A) Hematoxylin and eosin stain demonstrating replacement of tonsillar parenchyma by a dense infiltrate of mature-appearing plasma cells with eccentric nuclei and abundant eosinophilic cytoplasm, admixed with eosinophilic amorphous extracellular deposits consistent with amyloid. (B) CD138 immunohistochemical staining, demonstrating diffuse and strong membrane positivity, confirming the plasma cell nature of the infiltrate. (C) Lambda light chain immunohistochemical staining showing strong cytoplasmic positivity in the neoplastic plasma cell population. (D) Kappa light chain immunohistochemical staining showing negativity, establishing lambda light chain restriction and confirming clonality. (E) Congo red staining demonstrating extensive amorphous amyloid deposits within the tonsillar stroma, appearing salmon-pink under conventional light microscopy. (F) Characteristic apple-green birefringence of Congo red-stained amyloid deposits under polarized light microscopy, confirming the diagnosis of amyloidosis

Histopathological examination revealed a dense infiltrate of mature-appearing plasma cells exhibiting light-chain restriction. Immunohistochemistry demonstrated strong lambda positivity, kappa negativity, and CD138 positivity, consistent with a clonal plasma cell neoplasm. Congo red staining confirmed extensive amyloid deposition within the tonsillar stroma, displaying characteristic apple-green birefringence under polarized light. These findings correlated with the clinical presentation of a progressive, expansile tonsillar mass and the imaging features of a well-defined, noninvasive lesion, supporting a unified diagnosis of localized AL amyloidosis associated with a plasma cell neoplasm (Figure 4).

Following these findings, the patient was referred to the hematology service for further evaluation to exclude systemic amyloidosis. However, the patient declined additional diagnostic studies, which limited the ability to definitively classify the disease as localized or systemic.

## Discussion

Amyloidosis represents a diagnostically challenging entity due to its heterogeneous presentation and its ability to mimic more common conditions, including neoplastic processes [2,5]. In the head and neck region, this challenge is particularly evident, as lesions are often approached initially under the suspicion of malignancy.

In our case, the prolonged clinical course of approximately five years, combined with the absence of systemic symptoms, initially suggested a benign or indolent process. However, the progressive increase in size and extension toward the hypopharynx raised concern for a neoplastic lesion, which ultimately guided the decision for surgical management.

Localized amyloidosis is thought to result from local production of immunoglobulin light chains by a small population of plasma cells, which subsequently deposit in the surrounding tissue [1,4]. This mechanism explains both the localized nature of the disease and its frequent association with plasma cell infiltrates, as observed in our patient.

Although localized amyloidosis generally follows a more indolent course than systemic forms, distinguishing between these entities is critical. Systemic AL amyloidosis is associated with significant morbidity and mortality due to multiorgan involvement, and early diagnosis is essential to prevent irreversible organ damage [3,5].

In previously reported cases of tonsillar amyloidosis, patients are often asymptomatic or present with minimal symptoms, and lesions are frequently discovered incidentally [6,7]. In contrast, our patient presented with progressive symptoms over several years, which contributed to the clinical suspicion of malignancy and highlighted the variability in presentation.

Imaging studies in amyloidosis are typically nonspecific and may not reliably differentiate between benign and malignant processes. Therefore, histopathological examination remains the cornerstone of diagnosis. The identification of amyloid with Congo red staining, along with immunohistochemical characterization of light chain restriction, is essential for establishing both the diagnosis and the underlying subtype [2,3].

An important aspect of management is the need for a comprehensive systemic evaluation following histopathological diagnosis. This workup should include serum and urine protein electrophoresis, immunofixation, serum free light chain analysis, and bone marrow biopsy when clinically indicated. These studies are essential not only to exclude systemic AL amyloidosis but also to differentiate between two closely related yet prognostically distinct entities: localized AL amyloidosis with reactive plasma cell infiltration vs. extramedullary plasmacytoma with secondary amyloid deposition [8]. In the former, the plasma cell population is typically small and confined, with a generally favorable prognosis; in the latter, the plasma cell component constitutes the primary neoplastic process, with amyloid representing a secondary phenomenon, and the risk of progression to systemic disease or multiple myeloma is considerably higher [9].

In our case, the immunohistochemical profile, lambda-restricted CD138-positive plasma cells with extensive stromal amyloid deposition, is consistent with either entity, and definitive classification was not possible due to the patient's refusal of further diagnostic workup. This represents a critical diagnostic limitation that must be transparently acknowledged. Ideally, serum-free light chain ratio, bone marrow plasma cell percentage, and systemic imaging would have been obtained to complete the classification. Clinicians facing similar presentations should be aware of this distinction and counsel patients thoroughly regarding its implications for prognosis and follow-up.

Consequently, although the findings are highly suggestive of localized AL amyloidosis, the possibility of systemic involvement cannot be definitively excluded. This underscores the importance of patient counseling and multidisciplinary follow-up in similar cases.

## Conclusions

Localized amyloidosis of the palatine tonsil is an exceptionally rare entity that may clinically and radiologically mimic neoplastic disease. In cases of atypical tonsillar masses, especially those with a prolonged and indolent course, amyloidosis should be considered within the differential diagnosis.

Definitive diagnosis relies on histopathological evaluation, including Congo red staining and immunophenotyping to confirm amyloid deposition and characterize the underlying plasma cell population. Given the well-documented association between localized AL amyloidosis and clonal plasma cell proliferations, and the diagnostic importance of differentiating this entity from extramedullary plasmacytoma with secondary amyloid deposition, all patients should undergo comprehensive systemic evaluation comprising serum and urine electrophoresis, free light-chain analysis, and bone marrow biopsy when clinically indicated.

This case highlights the importance of integrating clinical judgment, surgical decision-making, and histopathological findings, as well as the need for multidisciplinary assessment in the evaluation of unusual oropharyngeal lesions.
